# Downregulation of TGF-beta receptor types II and III in oral squamous cell carcinoma and oral carcinoma-associated fibroblasts

**DOI:** 10.1186/1471-2407-11-88

**Published:** 2011-02-28

**Authors:** Wenxia Meng, Qingjie Xia, Lanyan Wu, Sixiu Chen, Xin He, Lin Zhang, Qinghong Gao, Hongmei Zhou

**Affiliations:** 1 State Key Laboratory of Oral Diseases, Sichuan University, Chengdu, Sichuan, China; 2 The Station Key Lab of Biotherapy, Sichuan University, Sichuan, China; 3 Department of Oral Pathology, West China Hospital of Stomatology, Sichuan University, Sichuan, China; 4 Department of Oral Oncology, West China Hospital of Stomatology, Sichuan University, Sichuan, China; 5 Department of Oral Medicine, West China Hospital of Stomatology, Sichuan University, Sichuan, China

## Abstract

**Background:**

The purpose of this study was to assess the expression levels for TβRI, TβRII, and TβRIII in epithelial layers of oral premalignant lesions (oral leukoplakia, OLK) and oral squamous cell carcinoma (OSCC), as well as in oral carcinoma-associated fibroblasts (CAFs), with the final goal of exploring the roles of various types of TβRs in carcinogenesis of oral mucosa.

**Methods:**

Normal oral tissues, OLK, and OSCC were obtained from 138 previously untreated patients. Seven primary human oral CAF lines and six primary normal fibroblast (NF) lines were established successfully via cell culture. The three receptors were detected using immunohistochemical (IHC), quantitative RT-PCR, and Western blot approaches.

**Results:**

IHC signals for TβRII and TβRIII in the epithelial layer decreased in tissue samples with increasing disease aggressiveness (P < 0.05); no expression differences were observed for TβRI, in OLK and OSCC (P > 0.05); and TβRII and TβRIII were significantly downregulated in CAFs compared with NFs, at the mRNA and protein levels (P < 0.05). Exogenous expression of TGF-β1 led to a remarkable decrease in the expression of TβRII and TβRIII in CAFs (P < 0.05).

**Conclusion:**

This study provides the first evidence that the loss of TβRII and TβRIII expression in oral epithelium and stroma is a common event in OSCC. The restoration of the expression of TβRII and TβRIII in oral cancerous tissues may represent a novel strategy for the treatment of oral carcinoma.

## Background

An increasing number of studies demonstrate that transforming growth factor beta (TGF-β) signaling pathway plays a dual role during the initiation and progression of human cancer; initially, it suppresses the formation of tumors, but elevated levels of TGF-β promote the growth, progression, and migration of established tumors. Different explanations have been proposed for this dichotomous function of TGF-β, including the possibility that TGF-β exerts tumor-suppressing effects on epithelial-derived tumor cells and tumor-promoting effects on stromal cells [1]. In general, TGF-β exerts its effect by binding to the TGF-β type II receptor (TβRII) and by subsequently recruiting TβRI for downstream cytoplasmic signaling via multiple parallel signaling pathways, including the SMAD proteins [2]. In addition, TβRIII functions as a coreceptor to increase the binding of ligands to TβRII. In a majority of human cancers and cell lines, the expression of TβRI and TβRII is altered at the protein and/or mRNA levels [3,4].

The in-depth study of TβRIII revealed that this receptor may have additional functions that are independent of ligand presentation. Ryan et al [5] showed that the expression of TβRIII (or betaglycan) is downregulated or lost in human prostate cancers compared with benign prostate tissues, at the mRNA and protein levels. Recently, another group used in vitro cell culture and in vivo animal models to demonstrate that the restoration of TβRIII expression in renal cell carcinoma resulted in a marked induction of apoptosis [6].

Human oral squamous cell carcinoma (OSCC) accounts for about 90% of malignant oral lesions. It is widely recognized as progressing in a multistep manner, with an initial presentation of premalignant lesions (among which oral leukoplakia (OLK) is the most common), and later development of hyperplasia and dysplasia, then in situ carcinoma, and finally invasive carcinoma [7]. However, the expression of the major components of the pathway, which include the systematic analysis of signaling receptors TβRI, TβRII, and TβRIII, remains intact in human OSCC [8,9]. The current study represents the first systematic analysis of the expression of TβRI, TβRII, and TβRIII during the process of oral epithelial carcinogenesis.

Recent studies strongly suggest that the clinical behavior of malignant tumors not only depends on alterations in the epithelial cells themselves, but is also affected by their interaction with the tumor-associated stroma. However, the components of these specialized stromal cells represent a complex network that includes inflammatory cells (lymphocytes, macrophages, and mast cells), activated fibroblasts, and cells comprising the vasculature. Therefore, it remains questionable whether one single factor will be the "magic bullet" of cancer therapy. Recent data bring prominence to the idea that activated fibroblasts (also termed carcinoma-associated fibroblasts (CAFs) or myofibroblasts) may be major players in the tumor stromal environment [10,11]. Their role as starting or supportive elements in carcinogenesis is also well established. Relevant studies of modified fibroblasts have been performed in several tumor systems [12,13]. Some of the systematic and important findings of our previous study suggest that oral CAFs promote the proliferation and invasion of the lingual carcinoma cell line Tca8113 in vitro by secreting the KGF (keratinocyte growth factor) and MMP-2 factors (matrix metalloproteinase) [14]. A recent study showed that TGF-β signaling in fibroblasts modulated the growth and oncogenic potential of adjacent epithelia in selected tissues [15]. Moreover, downregulation of TβRII was observed in colon-carcinoma-associated spindle-like stroma cells that apparently represented fibroblasts and myofibroblasts [14,16]. In our study, we directly separated and cultured oral CAFs in vitro from adjacent OSCC tissues and evaluated, for the first time, detected the expression levels of the three types of TβRs in oral CAFs.

## Methods

### Tissue Specimens

All surgically resected tissue specimens were obtained from the West China Hospital of Stomatology. For IHC analyses, 138 specimens representing four different clinicopathological stages (including 25 normal epithelium tissues, 21 OLK samples without dysplasia, 24 OLK samples with dysplasia, and 68 infiltrated OSCCs) were recruited from the archives of the Department of Oral Surgery and Oral Medicine. The 138 specimens included eight OSCC samples and paired normal tissues. The corresponding normal tissues were incised along a line that is surgically termed a safety border, which was verified via staining of frozen sections. All surgically resected tissues were collected, fixed in formalin, and embedded in paraffin for histopathological confirmation.

For primary cell cultivation, fresh and sterile tissue specimens (including OSCC and normal oral mucosal tissues) were placed immediately on Hanks Balanced Salt Solution (Gibco, USA) containing penicillin and streptomycin (200 μg/ml) after being collected from the patient. All samples contained the epithelium and adjacent connective tissues. The OSCC specimen was fresh and exhibited infiltration of the adjacent connective tissue. Connective tissues used for sectioning were collected as close as possible to the epithelium. The protocol was reviewed by the Institutional Ethics Committee of Sichuan University and informed consent was obtained from each patient.

### IHC Analysis

Immunostaining for TβRI and TβRII (rabbit polyclonal antibodies; dilution, 1:200; Santa Cruz, USA) and TβRIII (mouse monoclonal antibody; dilution, 1:200; Santa Cruz, USA) in human OSCC and tumor-associated stromal sections was performed using the DakoCytomation EnVision system (DakoCytomation Corporation, Carpinteria, CA), according to the manufacturer's instructions. Slides were counterstained with Modified Harris Hematoxylin. Immunoreactivity was scored as "-" (absent), "1+" (low, ≤ 25% of positive cells), "2+" (moderate, 26-75% of positive cells), or "3+" (diffuse, > 75% of positive cells) [9]. OSCCs were assessed based on at least 10 randomly selected fields. All slides were interpreted by two investigators.

### Isolation and Cultivation of Oral CAFs and NFs

The specimens were analyzed using methods described previously[14]. Briefly, tissues were washed twice with phosphate-buffered saline (PBS) and antibiotics. Oral epithelial and adipose tissues were then separated from surrounding stromal cells. The residual connective tissue was cut into small pieces (1 × 1 × 1 mm) that were maintained in Dulbecco's modified Eagle's medium (DMEM, Gibco, USA, pH 7.2) containing 20% fetal calf serum (Gibco, USA), glutamine (20 μg/ml), penicillin (100 U/ml), streptomycin (100 μg/ml), and 0.25% trypsinase (Gibco) at 37°C in an atmosphere containing 5% CO_2_. To purify the cells, we opted for a method of curettage combined with trypsinization, which was performed when cells covered the bottom of the culture bottle fully. Cultures at passage number three were used for the cellular identification of oral CAFs and NFs. A wide-spectrum α-cytokeratin antibody (ZSGB-BIO, Beijing, Corp., China) was used to confirm the absence of contaminating epithelial cells. Finally, antibodies against vimentin (ZSGB-BIO, Beijing, Corp., China) and smooth muscle α-actin (R&D Systems) were used to confirm the myofibroblastic nature of these cells [17].

### Quantitative Real-Time PCR

Total RNA was isolated from cells using the Trizol reagent (Invitrogen) and was quantified by analysis of absorbance at 260 nm. Relative gene expression levels were calculated using the comparative threshold-cycle method of quantitative PCR, with data normalized to β-actin and expressed relative to untreated controls. Real-time PCR was performed using the SYBR PrimeScript™RT-PCR kit II (TaKaRa, Dalian, China), according to the manufacturer's instructions. The primers used to amplify TβRI, TβRII, and TβRIII were as follows: TβRI sense, 5'-GGTCTTGCCCATCTTCACAT-3' and antisense, 5'-TCTGTGGCTGAATCATGTCT-3'; TβRII sense, 5'-GTCTACTCCATGGCTCTGGT-3' and antisense, 5'-ATCTGGATGCCCTGGTGGTT-3'; and TβRIII sense, 5'-TACAGAGAGAGGTCACACT-3' and antisense 5'-GTCTTCAGATGCCACACCAG-3'. The total volume used in PCR was 50 μL. PCR products (5 μl) were analyzed by electrophoresis using 1.5% agarose gels and were visualized by SYBR Gold (Molecular Probes, Eugene, USA) staining. All experiments were performed in triplicate.

### Protein Preparation and Western Blot Analysis

Protein extracts were prepared using a lysis buffer (RIPA) containing the protease inhibitor PMSF (RIPA:PMSF = 50:1). For Western blot analysis, ~80 μg of protein was separated using 8% SDS-PAGE, which was followed by transfer onto a PVDF membrane (Millipore, USA) and probing with an anti-TβR antibody (1:400, Santa Cruz, USA). The blots were incubated with horseradish-peroxidase-conjugated secondary antibodies (1:5,000) and visualized using an enhanced chemiluminescence detection system (Pierce Biotech Inc., Rockford, IL).

### Preparation of Cells Conditioned With the Human TGF-β1 Cytokine

To determine whether TGF-β1 mediates the regulation of the expression of TβRI, TβRII, and TβRIII, we assessed the levels of the TβRs using quantitative real-time PCR and Western blot analysis after treatment with TGF-β1.

TGF-β1 was obtained from Sigma (St. Louis, MO). CAFs or NFs cells were cultured for two consecutive days in medium containing 10% fetal calf serum and were then washed twice with PBS and cultured in serum-free medium containing 10 ng/ml of TGF-β1. After 24 h of treatment, cells were washed and harvested for RNA and protein extraction.

### Statistical Analyses

Differences between the groups were statistically evaluated using the unpaired Student's *t *test. The chi-squared or K Independent Samples Tests were used to compare frequencies, when appropriate. Significance was set at *P *< 0.05. All analyses were performed using the statistical software SPSS for Windows Version 13 (SPSS Inc., Chicago, USA).

## Results

### Expression of TβRI, TβRII, and TβRIII in Human OSCC, Oral Leukoplakia, and Normal Tissues

To fully assess the expression of TβRI, II, and III in clinical samples and determine their role in OSCC carcinogenesis and prognosis, 138 specimens were examined that represented four clinicopathological stages (including normal epithelium tissues, OLK without dysplasia, OLK with dysplasia, and infiltrated OSCCs). The clinical information of patients is shown in Table 1.

**Table 1:** Characteristics of the OSCC Tissue Samples Used for IHC Analysis

Age (years)	41-86
Sex	
Male	42
Female	26
Histological type	
Well diff.	25
Mod diff.	24
Poor diff.	19
T classification	
T1	19
T2	32
T3	6
T4	11
N classification	
N0	38
N+	30

**Figure 1:**
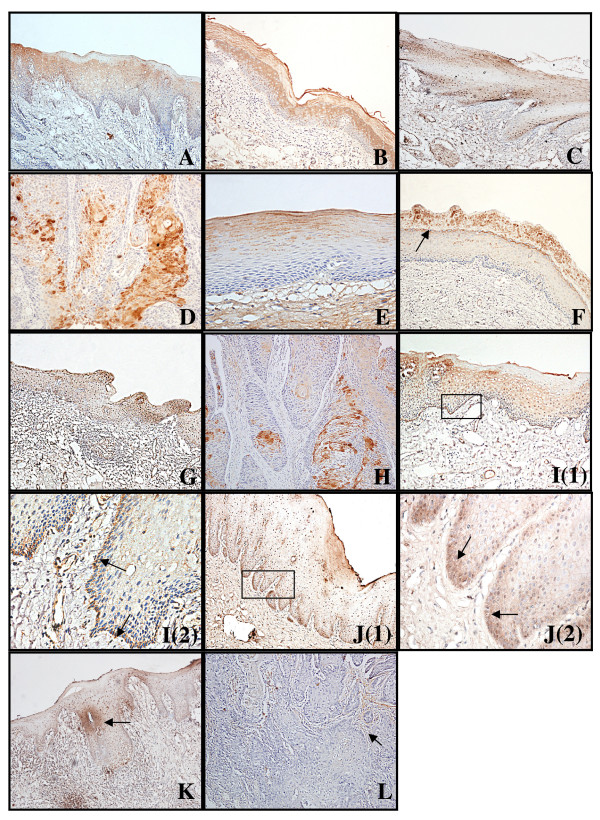
**The expression of TβRI, TβRII and TβRIII in human oral squamous carcinoma**. TβRI in OSCC tissues and in OLK showed the same homogeneous, moderate, and intense cytoplasmic expression as in normal oral specimens (A-D). IHC analysis showed that the expression of TβRII (E-H) and TβRIII (I-L) decreased gradually with the progression of carcinogenesis. (E) Strong signal for TβRII was detected in the upper spinous and granular layers of normal specimens. (F) Positive staining was detected mainly in the granular layers and in cuticular layer (arrowhead) in OLK without dysplasia. (G) The immunostaining profile was decreased in OLK with dysplasia. (H) Significant downregulation of TβRII was observed in infiltrated OSCC. (I) Strong signal for TβRIII was detected in the basal layer and in the upper spinous in normal specimens (arrowhead). (J) Positive staining was significantly decreased in OLK without dysplasia and a stronger signal for TβRIII was detected in the basal layer. (K) The immunostaining profile was decreased in OLK with dysplasia. (L) Note the absence of TβRIII staining in OSCC tissues. Weak expression of TβRIII was observed in OSCC stroma exclusively. (F) IHC scores for TβRI in patient-matched normal and OSCC samples.

**Figure 2:**
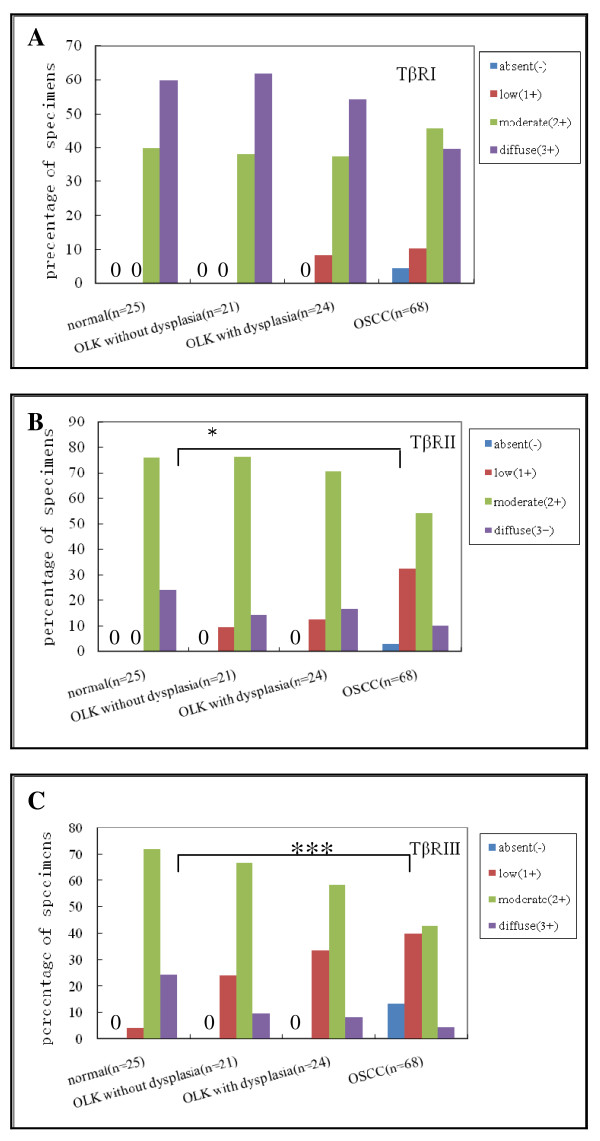
**Summary of IHC results, with percentages shown**. (A:TβRI) *P *= 0.116, K Independent Samples Tests. (B:TβRII) **P = 0.002, K Independent Samples Tests. (C: TβRIII) ***P < 0.001, K Independent Samples Tests. When staining is not detected, we stated in the figure (0).

**Figure 3:**
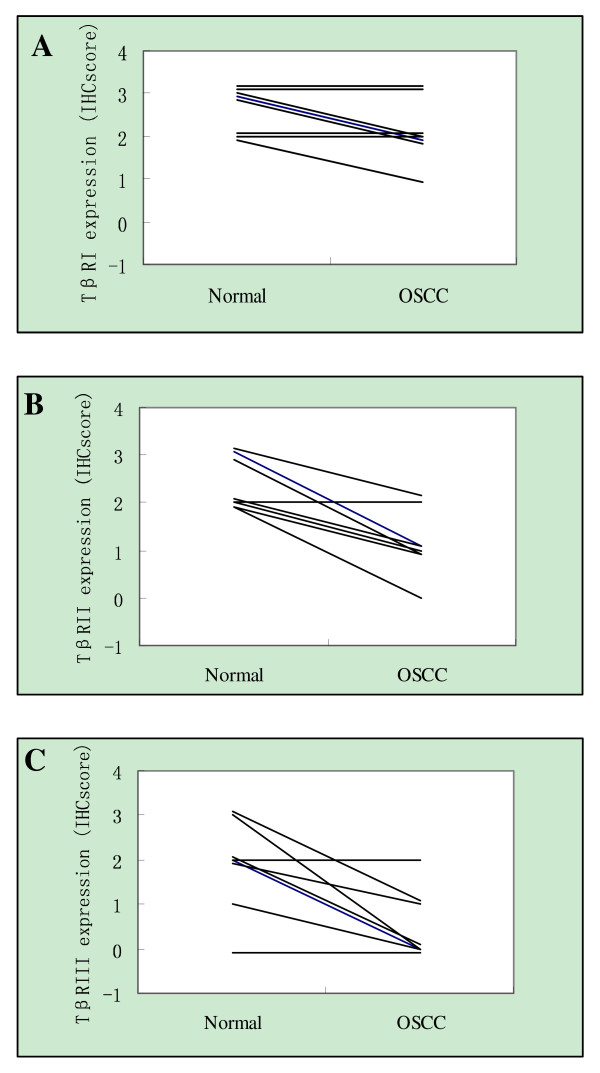
**IHC scores for TβRI, TβRII and TβRIII in patient-matched normal and OSCC samples**.

Our IHC results showed moderate-to-intense homogeneous cytoplasmic or membrane expression of TβRI, II, and III in oral normal squamous epithelium. Homogeneous, moderate, and intense cytoplasmic expression of TβR-I was observed in OSCC tissues and in OLK (Figure 1A-D; Figure 2A). However, TβRII and TβRIII staining in the four groups revealed a remarkable decrease in expression from normal oral specimens through OLK to OSCC (Figure 1E-L; Figure 2B and 2C), which was paralleled by an increase in the severity of epithelium dysplasia. Interestingly, we noted the upregulation of TβRIII at the basal layer, both in normal epithelium and oral leukoplakia (Figure 1 I(2), J(2)), whereas TβRII expression was nearly absent in the two oral epithelia. However, a strong signal for TβRII was detected in the upper spinous and granular layers of normal oral epithelium, as well as in the cuticular layer, especially in the OLK cases (Figure 1F). The proportion of cells exhibiting diffuse TβRII expression decreased from 24% in normal oral specimens to 10.3% in OSCC specimens. Concomitantly, the proportion of cells exhibiting low TβRII expression increased from 0% in normal oral specimens to 32.4% in OSCC specimens (*P *= 0.002, K Independent Samples Tests) (Figure 2B). The number of cells exhibiting diffuse TβRIII expression decreased from 24% in normal specimens to 9.5% in OLK without dysplasia and 8.3% in OLK with dysplasia, and to 4.4% in OSCC specimens. In contrast, the proportion of cells with low TβRIII expression increased from 4% in normal specimens to 23.8% in OLK without dysplasia and 33.3% in OLK with dysplasia, and to 39.7% in OSCC specimens (*P *< 0.001, K Independent Samples Tests) (Figure 2C). In addition, to address directly the role of the loss of TβR expression in OSCC progression, we assessed eight matched normal tissue samples and invasive OSCC specimens (Figure 3). The examination of TβRII expression revealed that six cases exhibited a decrease in expression, from relatively high levels (IHC score, 2-3) in normal oral tissues to low expression levels (IHC score, 0-1) in the matching invasive OSCC tissues (Figure 3B). TβRIII expression decreased from an IHC score of 2-3 in normal specimens to a score of 0 in OSCC specimens (Figure 3C). These data suggest that the expression of both TβRII and TβRIII was significantly downregulated in OSCC samples and that this loss of TβRII and TβRIII expression correlated with the development of OSCC (from normal tissues through OLK to OSCC).

### Identification of Oral CAFs and NFs

**Figure 4:**
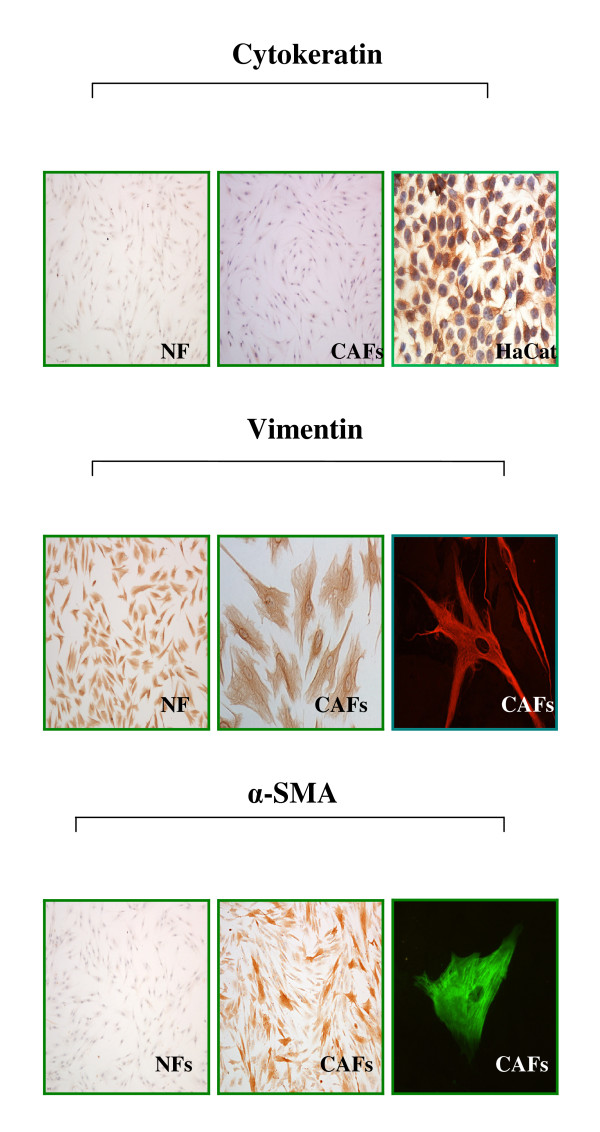
**Identification of oral CAFs and NFs**. The morphological characteristics of CAFs changed significantly compared with what was observed in NFs. CAFs showed positive staining for vimentin and α-SMA and were negative for cytokeratin. The positive control (human normal keratinocytes HaCat) is shown for cytokeratin stain.

Seven primary human oral CAF lines and six primary oral NF lines were derived from oral mucosa successfully using cell culture. The clinical information of the OSCC patients used for the establishment of primary CAF cultures is listed in Table 2. Normal tissues used for NF culture were collected from healthy individuals undergoing plastic or trauma surgery. We then verified the purity of the various fibroblast populations using immunostaining. These fibroblast populations were negative for cytokeratin, whereas they expressed fibroblastic marker vimentin, which suggests that some fibroblast traits were preserved in the CAFs (Figure 4). Importantly, cultured CAFs expressed traits of activated fibroblasts (myofibroblasts). Expression of alpha smooth muscle actin (α-SMA) is a defining characteristic of CAFs [10]. Using an anti-α-SMA antibody, we observed an increased proportion of α-SMA-positive myofibroblasts in isolated CAF populations (Figure 4) compared with normal fibroblasts.

**Table 2:** Clinical and Pathological Data of OSCC Samples Used for CAF Cultivation

Sample No	Sex	Age	Location	Histological type	TNM classification	Clinical stage
01	Female	38	Tongue	Well diff. SCC	T1N0M0	I

02	Female	57	Tongue	Well diff. SCC	T1N0M0	I

03	Female	64	Tongue	Mod. diff. SCC	T1N0M0	I

04	Male	50	Oral floor	Poor diff. SCC	T2N2M0	IV

05	Male	62	Gingival	Mod. diff. SCC	T2N2M0	II

06	Female	71	Gingival	Mod. diff. SCC	T2N2M0	II

07	Male	50	Buccal mucosa	Mod. diff. SCC	T1N0M0	I

### Loss of TβRII and TβRIII Expression in CAFs Assessed Using RT-PCR and Western Blotting

**Figure 5:**
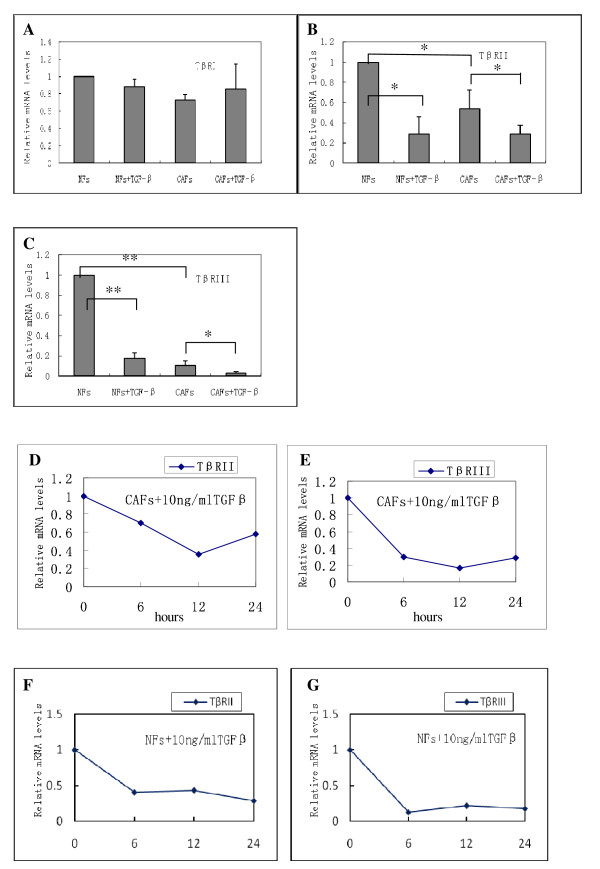
**Quantitative real-time PCR analysis of the mRNA levels of TβRI, TβRII, and TβRIII in CAFs and in CAFs in response to TGF-β1 (10 ng/ml) stimulation**. (A) The expression levels of TβRI were not significantly different in CAFs and NFs (*P *= 0.362); TGF-β1 treatment didn't change the TβRI levels in CAFs or in NFs. (B) TβRII mRNA expression was reduced in CAFs compared with NFs (*P = 0.004); The levels of expression of TβRII in oral CAFs or NFs were significantly decreased after treatment with 10 ng/ml TGF-β1 (*P = 0.0025). (C) TβRIII mRNA expression in CAFs was also significantly different from that of the control group (**P = 0.001). The levels of expression of TβRIII in oral CAFs or NFs were also significantly decreased after treatment with 10 ng/ml TGF-β1 (**P = 0.0035). (D, E) The maximal downregulation in oral CAFs with TGF-β1 was observed at 6-12 h. (F, G) The maximal downregulation in NFs with TGF-β1 was observed at 6 h.

**Figure 6:**
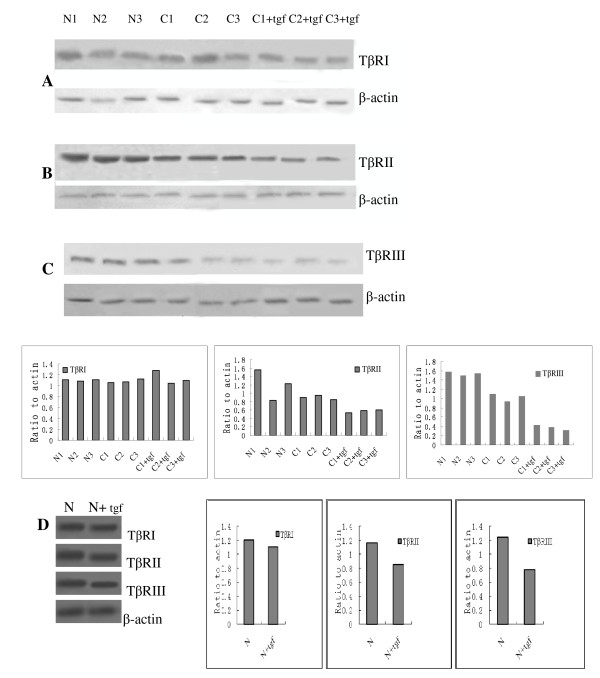
**Western blot analysis of the TβR proteins in NFs (N), CAFs (C), and in TGF-β-conditioned CAFs and NFs**. Western blot analysis revealed that the levels of expression of TβRI were increased in TGF-β-conditioned CAFs compared with untreated CAFs. The expression levels of TβRII and TβRIII were significantly decreased significantly in TGF-β-conditioned CAFs and TGF-β-conditioned NFs.

The mRNA expression levels of the three kinds of TβRs in CAFs and NFs were examined using quantitative real-time PCR. The expression of the TβRIII mRNA in CAFs was downregulated by a factor of 11.37 and was significantly different from the control group (*P *= 0.001). In addition, the expression of the TβRII mRNA was reduced by a factor of 2.013 in CAFs compared with NFs (*P *= 0.004). TβRI mRNA expression levels were not significantly different between CAFs and NFs (*P *= 0.362). Overall, the expression levels of the TβRI, TβRII, and TβRIII mRNAs, as assessed using RT-PCR analysis, were consistent with the immunocytochemistry results (Figure 5). Similarly, the expression of TβRII and TβRIII was decreased at the protein level in CAF cells compared with normal fibroblasts (Figure 6). No significant changes were observed regarding TβRI protein expression levels.

### TGF-β1 Downregulated the Expression of TβRII and TβRIII in Oral CAFs and in NFs

Our results showed that TβRIII and TβRII were significantly downregulated in oral CAFs or in NFs after treatment with 10 ng/ml TGF-β1, with a maximal downregulation at 6-12 h (Figure 5B-G). To assess whether the effects of TGF-β1 on the expression levels of TβRII and TβRIII were specific, we analyzed the role of TGF-β1 in the regulation of TβRI levels in oral CAFs. In contrast with the results obtained for TβRIII and TβRII, TGF-β1 treatment led to a slight increase in the levels of TβRI (this result was not significant) (Figure 5A). Besides, TGF-β1 could also downregulate the TβRIII and TβRII expression in NFs (Figure 6D). These results indicate that transcriptional and translational downregulation associated with increased expression of TGF-β1 in an oral cancer microenvironment (especially in CAFs) may represent a mechanism that leads to oral carcinogenesis. We found that TGF-β1 downregulated TβRII and TβRIII (and not only TβRIII) in oral CAFs and in NFs.

## Discussion

This study represents the first systematic demonstration of the downregulation of the TGF-β receptors TβRII and TβRIII in human OSCC, with approximately 35.3% of OSCC specimens demonstrating low or absent expression of TβRII and approximately 52.9% of specimens demonstrating low or absent expression of TβRIII. This decrease in the expression levels of TβRII and TβRIII correlated with the progression of OSCC. We also demonstrated that loss of TβRII and TβRIII expression was an early event that occurred initially during the OLK stage. However, there was no significant difference in TβRI expression between normal specimens and OLK or OSCC. Thus, we predict that loss of TβRII and TβRIII expression is the most common alteration in the TGF-β signaling pathway described in human OSCC. Studies performed on other tumor tissues regarding TGF-β receptors yielded similar results, and it has been described as having an association with carcinogenesis and tumorprogression [16,18,19]. Previous data showed TβRIII can enhance TGFβ-mediated inhibition of proliferation, invasion and angiogenesis. Reexpression of TβRIII in vivo in renal cell carcinoma models induced striking apoptosis [6]. These results were consistent with our current study of tumor progression of oral cancer cells in which TGF-β receptors were defective. The potential mechanism underlying these findings may be that the decreased expression of the two receptors leads to the decrease of TβRII- or TβRIII-mediated apoptosis. These studies, together with the present study, suggest a broad role for both TβRII or TβRIII as tumor suppressors in epithelial-derived malignancies.

Tumor occurrence and development are intrinsically correlated with the role of the surrounding stroma. In the current study, we demonstrated that TβRII and TβRIII played an important role in oral carcinogenesis. Did similar changes take place in tumor-associated stroma? Considering the complex composition of stroma, we chose to study CAFs as the object of our study, as these cells are considered crucial in the process of carcinogenesis. Carcinoma-associated fibroblasts (CAFs) have been regarded as a special kind of myofibroblasts, and they also were named as myofibroblasts in many reports [10,13,20,21]. The stromal microenvironment in human tumours play a key role in the formation of CAFs. This cell-type is mostly defined based on morphological characteristics, biological behavior (including promotion of tumor cell growth) [13] or the expression of markers such as a-smooth-muscle actin (a-SMA), fibroblast-activated protein (FAP), fibroblast-specific protein-1 (FSP1), neuron-glial antigen-2 (NG2) and PDGF β-receptor [10]. However, and a-SMA was mainly used to identify the CAFs [10,13,20,21]. In our study, the oral CAFs was also detected as expression of a-SMA. To verify our hypothesis, we first isolated and cultured seven primary CAF lines and then assessed the expression levels of TβRI, TβRII, and TβRIII. Coupled with the IHC observations from OSCC, both TβRII and TβRIII were downregulated frequently at the mRNA and protein levels in human oral CAFs compared with NFs. A more pronounced decline was observed for the expression of TβRIII compared with TβRII (11.37 fold vs 2.013 fold). Bacman D *et al *considered that the decrease of TGF-β receptor expression in stroma as in epithelial tumour tissue might occur via mutation or downregulation [16]. In addition, significant downregulation of TβRII and TβRIII was observed after treatment with TGF-β1 for varying periods. Although we observed a slight increase in TβRI expression, this observation was neither consistent nor significant. The mechanisms underlying the negative regulation mediated by TGF-β1 implicate primarily genes that regulate the cell cycle, including the downregulation of growth-promoting transcription factors, such as c-Myc, ID1, and ID2 [22]. TGF-β isoforms downregulate TβRIII in breast cancer cells; this effect was relatively specific for TβRIII, as it did not affect TβRI and TβRII [19,23]. However, here we demonstrated the loss of TβRIII and TβRII expression both in CAFs and in NFs after treatment with TGF-β1. The previous report together with our current discoveries lead us to conclude that loss of the expression of TβRIII and/or TβRII in CAFs is a mechanism that underlies the tumor-promoting function of TGF-β1. How might the changes in the cellular characteristics of CAFs, together with the loss of TβRII and TβRIII expression and their response to TGF-β1, affect oral carcinogenesis? The current results prompt us to hypothesize that the increase in the levels of expression of TGF-β during tumor progression stimulates the downregulation of TβRs, both in the tumor itself and in stroma (CAFs), which results in the TβR-mediated reduction of apoptosis. Accordingly, loss of functional TβRII and TβRIII signaling may contribute to apoptotic escape and tumorigenesis.

At present, three main strategies are used to target CAFs during cancer therapy: (a) targeting of the CAF signals that initiate or promote tumor growth, invasion, and metastasis; (b) targeting of the tumor signals that are responsible for the development of "adequate" tumor fibroblasts; and (c) elimination of the CAFs themselves, to abolish all interactions among heterotypic cell types [21]. However, the tumor-host interaction microenvironment is a large and complex network; therefore, it remains questionable whether the use of one single factor (such as antibodies) is efficient in cancer therapy. Two large randomized trials in SCLC failed to show any benefits for inhibitor treatment [24]. Thus, it is necessary to exploit novel strategies that use CAFs as targets in anticancer therapies. In the current study, we demonstrated that the expression of TβRII and TβRIII in oral epithelium and CAFs played an important role in tumorigenesis. In addition, the restoration of the expression levels of TβRII and TβRIII seems more feasible than prior strategies.

Taken together, these data suggest that the loss of the expression of TβRII and TβRIII in oral epithelium and stroma (CAFs) is a common event in OSCC patients. Therefore, we strongly believed that TβRII and/or TβRIII may be used as predictors of tumorigenesis and severity and represent adequate therapeutic targets. However, the understanding of the precise mechanisms involved falls short of what would be required for the development of practical clinical applications. Moreover, the restoration of the expression levels of TβRIII and TβRII in cancerous tissues of the oral mucosa may serve as a novel target for the treatment of oral carcinoma.

## Conclusions

The expression of both TβRII and TβRIII was significantly downregulated in oral epithelium and stroma (CAFs) in OSCC. Exogenous TGF-β1 could downregulate both TβRII and TβRIII in oral CAFs and in NFs. Moreover, the restoration of the expression levels of TβRIII and TβRII in cancerous tissues of the oral mucosa may serve as a novel target for the treatment of oral carcinoma.

## Competing interests

The authors declare that they have no competing interests.

## Authors' contributions

WXM carried out the experimental studies and drafted and completed the manuscript. QJX participated in the design of the study and performed the statistical analysis. LYW, SXC, XH and LZ participated the tissue collected and tumor pathological characteristics. HMZ and QHG conceived of the study and participated in the design and coordination as well as helped to draft the manuscript. All authors read and approved the final manuscript.

## Pre-publication history

The pre-publication history for this paper can be accessed here:

http://www.biomedcentral.com/1471-2407/11/88/prepub
